# On the Stability and Abundance of Single Walled Carbon Nanotubes

**DOI:** 10.1038/srep16850

**Published:** 2015-11-19

**Authors:** Daniel Hedman, Hamid Reza Barzegar, Arne Rosén, Thomas Wågberg, J. Andreas Larsson

**Affiliations:** 1 Applied Physics, Division of Materials Science, Department of Engineering Sciences and Mathematics, Luleå University of Technology, SE-971 87 Luleå, Sweden; 2 Department of Physics, Umeå University, SE-901 87 Umeå, Sweden; 3 Department of Physics, University of Gothenburg, SE-412 96 Göteborg, Sweden; 4 Department of Physics, University of California and the Lawrence Berkeley National Laboratory, Berkeley CA 94720, USA

## Abstract

Many nanotechnological applications, using single-walled carbon nanotubes (SWNTs), are only possible with a uniform product. Thus, direct control over the product during chemical vapor deposition (CVD) growth of SWNT is desirable, and much effort has been made towards the ultimate goal of chirality-controlled growth of SWNTs. We have used density functional theory (DFT) to compute the stability of SWNT fragments of all chiralities in the series representing the targeted products for such applications, which we compare to the chiralities of the actual CVD products from all properly analyzed experiments. From this comparison we find that in 84% of the cases the experimental product represents chiralities among the most stable SWNT fragments (within 0.2 eV) from the computations. Our analysis shows that the diameter of the SWNT product is governed by the well-known relation to size of the catalytic nanoparticles, and the specific chirality is normally determined by the product’s relative stability, suggesting thermodynamic control at the early stage of product formation. Based on our findings, we discuss the effect of other experimental parameters on the chirality of the product. Furthermore, we highlight the possibility to produce any tube chirality in the context of recent published work on seeded-controlled growth.

Single-walled carbon nanotubes (SWNTs) possess remarkable electrical, mechanical and chemical properties, and are widely discussed for various applications such as integration in electronic circuits^1^,^2^,^3^, mechanical reinforcement of light-weight composites^4^,^5^, as well as for chemical- and bio-sensors^6^,^7^ and synthetic muscles^8^. However, many applications rely on having SWNTs with defined properties and, since both electrical and structural characteristics of SWNTs can vary greatly depending on the chirality and diameter, huge efforts have been made to either selectively grow SWNT with single or few chiralities^9^,^10^,^11^,^12^,^13^,^14^,^15^,^16^,^17^,^18^,^19^,^20^,^21^ or to post-purify them after synthesis^22^,^23^,^24^,^25^,^26^,^27^,^28^,^29^,^30^,^31^. Alternatively, efforts have been made to tailor SWNT properties by chemical doping^32^,^33^,^34^,^35^,^36^,^37^,^38^,^39^,^40^,^41^,^42^, which significantly affects the electrical and chemical properties of SWNTs.

Although the post-purification methods result in relatively high yield of certain chiralities; such methods are expensive and time consuming, and may modify the properties of the tube, for example, by functionalization or structural damage. The chemical doping approach, on the other hand, suffers from the fact that the dopant elements can incorporate into SWNTs in different configurations; therefore doped samples are usually inhomogeneous. The limitations and disadvantages of the post modification methods have encouraged researchers to continue investigating possible approaches to selectively grow SWNTs. Chemical vapor deposition (CVD) has become the most promising technique for this purpose, since it involves several controllable growth parameters. The vapor-liquid-solid^43^,^44^,^45^ (VLS) mechanism of fiber growth serves as a basis for understanding SWNT growth through catalytic-CVD, but one also has to account for the tubes being hollow: Since this results in an unstable growing end that has to be stabilized by a catalyst particle that has the metal–carbon binding energy in the required region–not too weak and not too strong, following a “Goldilocks” principle that is only fulfilled for a limited number of metals^46^,^47^,^48^,^49^, and can be tuned by alloying^50^,^51^. It has been shown that it is possible to affect the chirality of the growing SWNT by varying the experimental conditions, such as catalyst composition^12^,^14^,^15^,^16^,^52^,^53^,^54^,^55^ temperature^56^, carbon precursor^10^,^17^, carrier gas pressure^57^, and catalyst support^11^,^18^. Concurrently, several reports have investigated the energetic stability of different chiralities^58^,^59^ and their caps^60^,^61^,^62^,^63^, and some studies have also compared theoretical and experimental data in an effort to clarify the production of SWNTs of certain chiralities^64^,^65^. However, despite numerous studies on the production of SWNTs, a detailed understanding of the correlation between experiment and theory is lacking. In our study we try to answer the following key questions; i) To what extent can the observed statistical abundance of certain SWNT chiralities be explained by their energetic stability?, ii) What experimental growth parameters have high impact on the chirality of the growing SWNT?, iii) Why are certain tubes so seldom observed in experimental studies? and, finally, iv) What strategy should be followed to obtain a high relative abundance of more exotic tubes seldom seen in SWNT products?

We try to answer these questions by taking advantage of all relevant experimental studies made so far, meaning that we consider experiments where methods have been used that are able to properly detect all or nearly all present SWNT chiralities in a sample with tolerable statistics. SWNTs with different chiralities and diameters all have unique band structures, which influences the criteria for eligible detection techniques. For example, photo-luminescence only detects semiconductor SWNTs and in the case of Raman spectroscopy it is necessary to have excitation wavelengths equal to the difference between van Hove singularity (VHS) pairs to satisfy the resonance condition^66^,^67^,^68^. In the literature survey made for our comparison of theoretical stability and experimental abundance we have defined three main techniques that we deem to be appropriate to meet this criterion; either a combination of photo-luminescence, electron diffraction, and/or ultraviolet-visible-near infrared spectroscopy or Raman spectroscopy obtained by at least three different excitation lasers evenly distributed over the wavelength region from 450–1054 nm (1.2–2.8 eV). A single study that utilizes surface enhanced Raman spectroscopy is also considered in the study, as it seems to properly detect all present SWNTs^69^. There is a risk that even in the selected studies some tubes might be out of resonance with the excitation wavelength used for detection, but since our statistics are based on number of “hits” and not the statistical abundance, and since each resonance window for a SWNT is roughly 0.3 eV^70^, we believe that our criterion gives valid data for our analysis. The data from the experimental studies are collected in Table S1 in the supplementary material. The combined experimental data are discussed in the context of theoretical data on the energetic stability of eleven different SWNT-series, namely the (n + m = 8) to (n + m = 18) series, where n and m are the SWNT chiral indices.

## Methods

**Figure 1:**
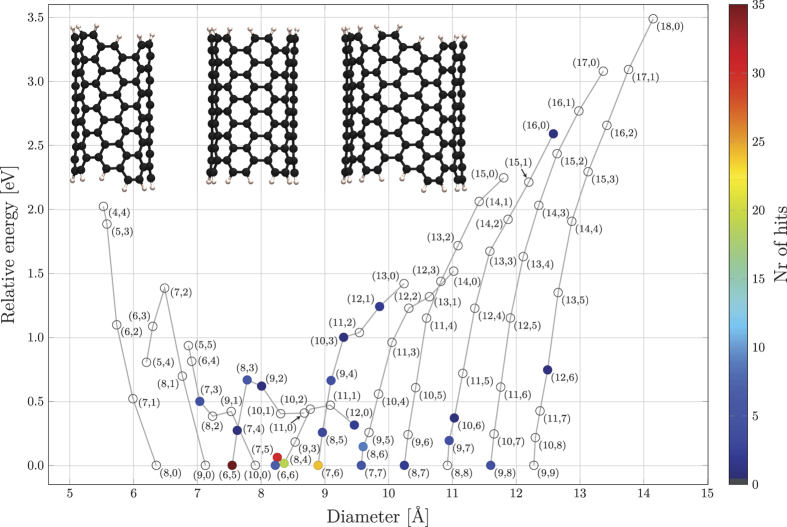
Relative energies between SWNTs of each (n + m) series from (n + m) = 8 to 18 plotted against the nanotube segment diameter. The zero line thus represents the most stable tube in each series. The colored points represent products from SWNT growth, with the color code representing the hit-rate of how many times a particular SWNT has been reported as a product in a unique CVD experiment (see Table S1). Inset shows the hydrogen terminated six-layer SWNT segments of the (6,5), (6,6) and (9,8) armchair and near-armchair tubes.

We have calculated the total energy of all carbon nanotube series for which (*n* + *m*) = 8, 9, 10,..., 18 using density functional theory (DFT). The nanotubes were modeled using six-layered hydrogen terminated structures, for which the number of carbon atoms per layer is equal to 2(*n* + *m*) (see inset to Fig. 1). These structures were placed in a 27 × 27 × 27 Å cubic box, giving at least 10 Å of vacuum separation between the periodic images.

The DFT calculations were performed using the Vienna Ab initio Simulation Package^71^ (VASP). Only the gamma point was used in the calculations to minimize the interactions between periodic images. The calculations were performed using the projector-augmented wave method^72^, a plane wave basis set and the Perdew-Burke-Ernzerhof (PBE) exchange-correlation functional^73^. The plane wave basis set energy cutoff was set to 575 eV and the electronic self-consistence loop was converged to 1 × 10^−6^ eV.

Gaussian smearing was used; the smearing width (SIGMA) was adjusted in order to give energies associated with the electronic entropy of approximately 0.5 meV/atom. All of the calculations were spin polarized (ISPIN = 2), with an antiferromagnetic initial magnetization (one end of the SWNT spin up and the other spin down). The nanotube structures were relaxed with no symmetry constraints using the conjugate-gradient algorithm, until all of the forces acting on the ions were smaller than 6 × 10^−3^ eV/Å.

The formation enthalpy *H_f_* was calculated using a simplified version of the equation given in^74^


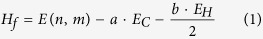


Here, *E*(*n,m*) is the energy of the SWNT, *E*_*C*_ = −9.201514 eV is the energy per C atom for a 500 atom super-cell of graphite and *E*_*H*_ *=* *−*6.771933 eV is the energy for an H_2_ molecule in a 27 × 27 × 27 Å cubic box. *a* *= *6 × 2(*n* + *m*) is equal to the number of carbon atoms in the nanotube and *b* = 2(*n* + *m*) is the number of hydrogen atoms used for termination of the nanotube ends. The diameters of the SWNTs were determined using the relaxed structures through the radial distance to the center-line of the tube averaged over the 4 central layers (excluding the hydrogen atoms and one layer at each side of the tube).

## Results and Discussion

We have computed the total energies of six-layer SWNT segments of the (n + m = 8) to (n + m = 18) series with hydrogen-terminated ends (see inset to Fig. 1). The relative energies of the segments within each series are shown in Fig. 1, meaning that the most stable tube in each series corresponds to zero energy. As reported in earlier studies, hydrogen-terminated tube segments with a zigzag character have antiferromagnetic ground states^75^,^76^,^77^. We report energy windows (∆E) of the difference between the most stable and least stable SWNT within the series (see Table 1) in agreement with previously reported values for the 9 to 11 series^58^, and the 12 and 13 series^59^.

In the lower series 8 to 10 zigzag tubes are the most stable while in the other series (11 to 18) armchair tubes are the most stable. In the 13-series and above the stability follows the chiral index, with the zigzag tubes being the least stable within each series. In the 8 series the stability follows the reverse order, with the armchair tube being the least stable. In the 9 series the order of stability is (9, 0), (8, 1), (5, 4), (6, 3), and (7, 2). In the 10 series the order is (10, 0), (8, 2), (9, 1), (7, 3), (6, 4), and (5, 5), and in the 11-series it is (6, 5), (7, 4), (10, 1) and (11, 0) only differing with 5 meV, (9, 2) and (8, 3). In the 12-series the order is (6, 6), (8, 4), (7, 5), (9, 3) (12, 0), (10, 2) and (11, 1).

We have reviewed the reported SWNTs in all relevant experimental studies^10^,^11^,^12^,^14^,^15^,^16^,^17^,^18^,^20^,^21^,^52^,^53^,^54^,^55^,^56^,^57^,^69^,^78^,^79^,^80^,^81^,^82^,^83^,^84^,^85^,^86^,^87^,^88^,^89^, which properly detect all present SWNT chiralities in the sample, and correlate the resulting statistics with the computed relative stabilities in Fig. 1. Specific CVD conditions are tabulated in Table S1 in the supplementary material.

**Figure 2:**
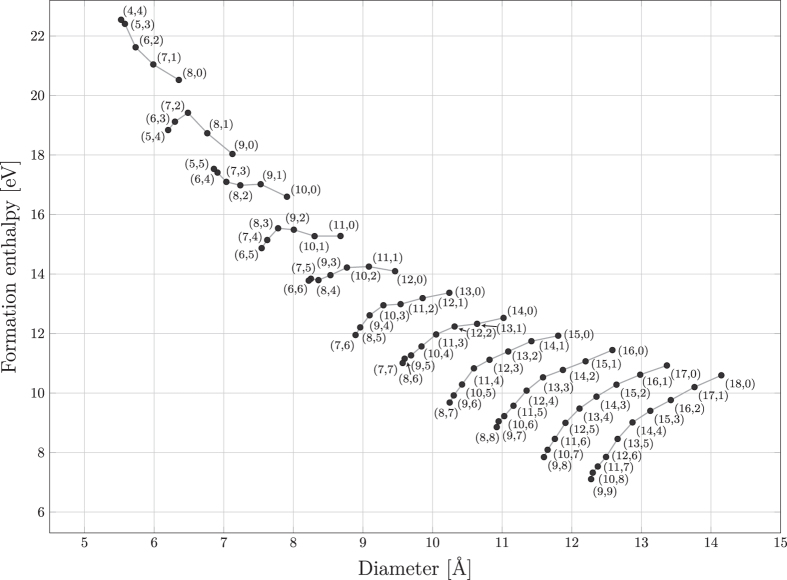
Formation enthalpy of each SWNT in this study (as defined by Eq. (1) in the main text) plotted against the nanotube segment diameter. This figure thus displays the energies of the tubes on a comparable scale.

Four observations can be made from this rather simplistic experimental/theoretical comparison: Firstly, there are no reported SWNT products in the 8 and 9 series (and only (7,3) from the 10 series), which we attribute to the fact that these small-diameter tubes have formation enthalpies that are too large (see Fig. 2). Secondly, most of the products are in the 10 to 14 series, although the formation enthalpies of the higher series are progressively lower. We argue that this is the result of the catalyst particle size targeted in the majority of these studies, as there is plenty of evidence for the relationship between catalytic metal nanoparticle size and the diameter of the grown tubes^20^,^86^,^90^,^91^. Thirdly, and most importantly, we observe that the vast majority of all SWNT products found in the experimental reports are among the most stable tubes in its series. This is a very surprising finding, since the energy differences between different chiralities are rather small, especially considering the differences in stability between the different series (see the formation enthalpies in Fig. 2)^92^,^93^. We have thus found that if no special measures are taken, the majority of the grown SWNTs will correspond to the few most stable SWNTs within the series, under the condition that the nanotube diameter matches the size of the metal nanoparticles used to catalyze the growth of the SWNT. Fourthly, we note, however, that there seems to be one major exception to the strong correlation between abundance and energetic stability; All experimental data indicate significantly lower abundance of pure armchair tubes (n=m) compared to near-armchair tubes (n ≈ m), despite the fact that armchair tubes display higher energetic stability, especially for series 12 and higher (see values for (6,6), (7,7), (8,8) and (9,9) in Fig. 1). The explanation for this can be found in the work by Yakobson group, who report that pure-armchair tubes (and pure-zigzag tubes) grow by a different growth mechanism than all other (so-called chiral) tubes that grow from screw-dislocations^64^,^65^. As a result, armchair and zigzag tubes will grow significantly more slowly (and hence be shorter) than chiral tubes. Since spectroscopic techniques such as Raman spectroscopy and photoluminescence scale with number of phonons, and number of electrons in the VHS, respectively, they both scale with number of atoms in the SWNTs, and hence an equal abundance of e.g. the armchair (6,6) and the chiral (7,5) tube in a given experiment will display a weaker signal of the armchair tube, since these have a shorter length^94^. It is important to point out that this observation does not rule out the possibility to target pure-armchair tubes as the main product, as recently shown by Sanchez-Valencia *et al.*^89^, since when using synthesized molecules as seeds (as discussed later) such tubes can indeed dominate. We note that our computational data points toward the (10,0) zigzag tube being produced in CVD growth using small diameter catalytic particles, but their short lengths make them undetectable.

We have found that the majority (84%) of the experimental product (hit-rate in Fig. 1) is among the most stable SWNT fragments (below 0.2 eV). Looking at tubes with similar diameter it is clear that, in addition to the relative energies of the tubes within the same series, the differences in formation enthalpy (see Fig. 2) is essential in understanding what SWNTs are formed: Clear examples where the formation enthalpy plays a major role in the growth abundance of certain chiralities can be seen by comparing e.g., the (7,5) and (8,4) tubes of the 12 series that both have significantly higher “hit-rates” than the (10,1) tube of the 11 series that have similar diameter as the (7,5) and the (8,4) tubes but a formation enthalpy that is around 2 eV higher. Similar observations can be made for the abundant (7,7) and (8,6) tubes of the 14 series and the rarely seen (10,3) and (11,2) tubes of the 13 series with similar diameters but much higher formation enthalpy. The above analysis of the data in Fig. 2 shows comprehensively how the experimental hits are concentrated near the baseline when recasting the data into series disregarding the stability relation between different series, as in Fig. 1. Lastly, we want to point out the rather remarkable fact that we have been able to draw all these conclusions using just SWNT segments, *without considering* their interface with the catalytic metal.

### Growth parameters discussion

In the context of our findings that the catalyst particle size together with the relative energetic stability represent the two most important criteria that influence the growth of specific SWNT chiralities, we review the effects of the other growth parameters, temperature, pressure, feedstock, substrate and catalyst composition, on the selective growth of SWNTs.

### Temperature

**Figure 3:**
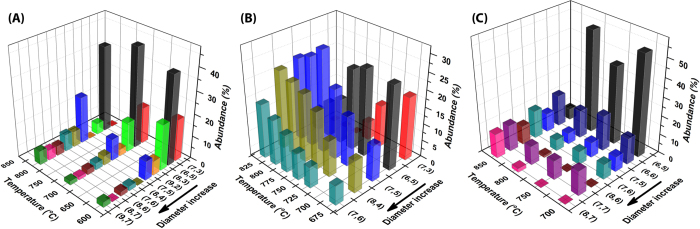
Effect of temperature on chirality distribution (**a**) represented from work by Loebick *et al.*^86^ (**b**) represented from work by Wei *et al.*^85^ (**c**) represented from work by Lolli *et al.*^56^. The arrows indicate the direction of diameter increase.

The most distinct impact that temperature has on the growth products is the increase in overall tube diameters. This is obvious from Fig. 3a–c. The increase in diameter can be rationalized by an effect on the catalytic metal nanoparticles known as Ostwald ripening^95^,^96^,^97^,^98^ at temperatures above 700 °C, meaning that the metal atoms become more mobile when the temperature rises. The result is disappearance of small metal nanoparticles where the material builds up the size of the remaining particles, as well as other temperature effects, such as agglomeration. This is exemplified by the report by Loebick *et al.*^86^, who showed that the average size of Co nanoparticles increases with temperature in the CVD reaction chamber. Although, in their work, the Co nanoparticles were deposited on MCM-41 mesoporous silica template and mixed with Mn to minimize the effect of temperature, still, the average diameter of the catalyst particles increases slightly at higher temperature, namely from 8 Å at 600 °C to 12.5 Å at 800 °C (at intermediate temperature, 700 °C, the particle size increases to 9 Å). As a result, the dominating SWNTs in the sample are also shifted, as represented in Fig. 3a, showing the chirality distribution *vs* growth temperature. Further, it is interesting to note that production of tubes with high relative energy for the diameters represented by the metal particle size distribution in this specific experiment, such as the (7,3), (8,3) and (9,2), is shifted to production of tubes with lower relative energy such as (8,4), (8,6), and (9,7) (*cf*. Fig. 1). This phenomenon will be further discussed below. The change in particle size (tube diameter) with temperature seems to be more significant in smooth (nonporous, weakly interacting) substrates such as silicon wafer. For instance, Fouquet *et al.*^14^ observed an up-shift in diameter distribution of the grown SWNT from 6.1–11.9 Å for 600 °C (growth temperature) to 6.3–14.9 Å for 700 °C, for Co catalyst deposited on a silicon wafer. The temperature increase also shifts the dominant chiralities from (6,5), (6,6) and (7,4) for 600 °C to (7,5), (7,6) and (10,9) tube for 700 °C. These observations manifest our previous statement about the strong correlation between relative energetic stability and high abundance under the pre-requirement of matching catalyst particle size since, although these tubes belong to different series with different formation enthalpies (see Fig. 2), the “new” observed tubes also have the lowest energies in each series but with larger diameters (see Fig. 1). Figure 3b,c show two other examples of the temperature effect: Wei *et al.*^85^ grew SWNTs by plasma-enhanced ethanol decomposition using Co catalyst particles supported on MCM41, while Lolli *et al.*^56^ studied the temperature effect for SWNT growth on CoMo catalysts. In our report we have given a few representative examples to reveal the effect of temperature on the selective growth process by highlighting studies that have examined the chirality distribution in detail. Our conclusions are, however, perfectly valid when compared with the data in other work^16^,^80^,^81^,^82^.

### Pressure

We also consider the carbon precursor pressure in the reaction chamber, which has been shown to have a significant effect on the chirality distribution. The influence of this parameter is, however, more complex to study, since it may also relate to the composition of the catalyst particles, with regard to their carbon saturation, especially at elevated temperature. Picher *et al.*^99^ showed that a combination of precursor pressure and reaction temperature affects the chirality distribution of the growing SWNTs and suggest that, compared to large catalyst particles, small catalyst particles can withstand higher carbon content before saturation. As a consequence, at a specific temperature, higher carbon precursor pressure will result in preferential activation of small catalyst particles and, consequently, preferential growth of small diameter tubes. A similar conclusion was made by Wang *et al.*^57^, who studied carbon precursor pressure ranging from 2 to 18 bar at fixed reaction temperature.

### Feedstock

Figure. 3a,b also introduce another important parameter for SWNT growth: the choice of carbon precursor. While Loebick *et al.*^86^ used CO (Fig. 3a) as carbon precursor, Wei *et al.*^85^ used ethanol. Several studies have speculated that the hydrogen produced when using a hydrocarbon as precursor affects the rate of catalyst particle reduction, remaining active for a longer time^56^,^100^,^101^. This is consistent with the results presented in Fig. 3a,b, showing a distribution with more small-diameter tubes for the CO precursor. Similar results have also been observed in other reports^99^. Wang *et al.*^10^, examined four different carbon precursors; CO, C_2_H_2_, C_2_H_5_OH and CH_3_OH to grow SWNTs and they also observed a clear shift to large-diameter tubes when hydrocarbons were used compared to CO.

### Substrate

The studies presented in Fig. 3a,b utilize CoMn and Co catalyst particles deposited on MCM41. Here, it is interesting to note that Mn is highly stable against reduction and does not act as a catalyst for SWNT growth (too large M-C bond strength)^49^, but instead acts in the alloy to prevent melting and evaporation of Co from the particles, thereby minimizing the effect of Ostwald ripening and agglomeration. This is the reason that the chirality distribution is less affected by temperature in Fig. 3a. For the same reason, inclusion of other non-catalytic metals (e.g., Mo, W, Cr, etc.) into the catalytic particles has a positive effect, producing uniform distributions of SWNTs, most likely because they help to reduce the effect of temperature^10^,^19^,^55^. It should be noted that inclusion of these metals also affects the catalytic particles’ effective M-C bond strength^50^,^51^. We also find that at low temperature these alloy catalysts lead to production of tubes with higher relative energy for the diameters represented by the metal particle size distribution in this specific experiment ((7,3), (8,3) and (9,2) in Fig. 3a). The reason for this is that the surface geometry of the particles does not adapt, but remains frozen (see more about this effect below). These observations also relate to the very important relation between the catalyst particles and the substrates for SWNT growth. Such particle-substrate interaction can also prevent restructuring of the particles driven by temperature as well as affecting the morphology of catalyst particles. Lolli *et al.*^56^ used CoMo supported on two different materials, SiO_2_ and MgO, to grow SWNTs under similar reaction conditions. Interestingly, the chirality distribution was broader when MgO was used as support, which the authors explain by a difference in particle-substrate interaction as well as a change in the catalyst particle morphology. Thus, the choice of substrate can have an effect on the restructuring of the catalytic particles similar to that of the alloying elements Mn, Mo, W and Cr, which will be considered below.

### Catalyst composition

Another parameter that affects the chirality distribution is the interaction between catalyst particle and the dissociating carbon atoms. Within the M-C bond strength “Goldilocks” window of catalytic activity^46^,^47^,^48^,^49^ (see Introduction), the use of different metals with different bond strengths affects the distribution of SWNT product. This effect has been described by Barzegar *et al.*^20^, who show that slight changes in the Co/Fe ratio of the catalyst particles could influence the chirality distribution. Similarly, H. Chiang *et al.*^53^ observed a change in chirality distribution of the grown SWNTs when they systematically studied a change in catalyst particle composition from pure Fe to pure Ni (with a number of steps utilizing different admixtures of Fe and Ni), while the average catalyst particle size was kept constant.

Overall the experimental and theoretical data strongly suggest that a selective growth mechanism is dictated by a *two-parameter model* in which the *diameter* of the tubes produced is primarily governed by the nature of the catalyst particle, in particular size, and the *specific chirality* is governed by the relative energetic stability at the diameter in question, pointing towards a high degree of thermodynamic control at the initial stage of growth. There are a few exceptions to the two-parameter model. In some cases a relatively high abundance of tubes with lower stability, such as the (7,3), (8,3), (9,2), and the (9,4) tubes, can be found. We have described above that in such cases the conditions to grow “low-stability” tubes occur for SWNT growth at low temperatures. It is reasonable to think that certain metal nanoparticles with ideal size and surface morphology can catalyze the growth of such tubes and that the low temperature hinders adaptation of the nanoparticles to the geometry demanded for more stable tubes with this diameter.

### Seeded growth

We subsequently turn our focus to special measures that can be taken in order to promote the growth of energetically unfavorable SWNTs found higher up in the ladder of a series. Two recent reports with very different seeding approaches give important insight into this matter: Sanchez-Valencia *et al.*^89^ use pre-grown chemically synthesized precursors of (6,6) caped segments as seeds to promote the growth of single chirality SWNTs samples. This work is in some aspects inspired by the work by Smalley *et al.*^102^, who use pre-cut SWNT segments as seeds to promote single chirality growth; Yang *et al.*^69^,^103^, on the other hand, take in two different studies advantage of a metal catalyst with a very high melting temperature, W_6_Co_7_. By growing nanotubes at 1030 °C a nearly complete selective growth process is achieved, with a 94% selectivity for the (12,6) tube, and a 80% selectivity for the (16,0) tube, respectively. Looking at Fig. 1, it is clear that the (12,6) tube is far from being the most stable tube in the 18 series. The authors explain the stabilization of this specific tube with the very high melting temperature of W_6_Co_7_ (2500 °C), which results in an unchanged morphology of the faceted catalyst nanoparticles during the CVD growth. The authors also indicate that, under slightly different conditions, other SWNTs with high relative energy for their diameter (as found in the present study, see Fig. 1) can grow, such as (14,4). The hypothesis made by the authors about frozen catalyst surfaces giving preference to certain tubes is fully supported by our observations. Tubes with high relative energy can grow from catalyst nanoparticles that exactly meet the requirement of only this specific chirality when frozen. If the conditions allow, the catalyst nanoparticle will adapt to the shape (and size) that better match the tubes with higher stability, but at low temperature (as discussed previously), for very stable catalyst particles (for example, due to high melting temperature), or for catalyst nanoparticles that are stabilized due to other reasons, exotic low-stability tubes with certain chirality can grow. The latter proposal is supported by our analysis of the experimental data, summarized in Fig. 1, which for some reports indicate a rather high abundance of e.g., the (7,4), (9,2) and (8,3) tubes of the 11 series, and the (8,5), (9,4) and (10,3) tubes of the 13 series. Looking further in Table S1, it can be seen that, in all of these experiments, the catalytic metal particles are deposited on a porous substrate that forms strong bonds to the metal catalyst. We believe that using such substrates results in a broader catalyst particle distribution and that the strong bonds between the particles and the substrate inhibit both reshaping of metal nanoparticles as well as diffusion of metal atoms, resulting in larger particles, thus providing a template for less stable tubes to be formed. The molecular seeds used in the Sanchez-Valencia *et al.*^89^ study, on the other hand, represent a very promising development where cap, ring and belt-shaped molecules from chemical synthesis (such as the molecules in ref. 104) could be used to promote the growth of particular SWNTs in a controlled way (as also suggested in ref. 73). Such seeds could also be substrate bound (non-catalytic) with subsequent deposition of the catalyst metal in order to reap the benefits of tip growth, both for feedstock access (SWNT length) and closely packed seeds (SWNT packing density). Bear in mind that the tube with the highest formation enthalpy seen in CVD is the (7,3) tube and, thus, *all tubes with lower formation enthalpy (see* Fig. 2*) could be possible products* that could be targeted through seeded growth. The molecular seeding approach can also be used to eliminate the complication of using metal nanoparticles and the tube diameter dependence on their size, since the (6,6) tube growth in ref. 89 was catalyzed by a smooth Pt surface.

We believe that the major effect of the growth temperature, of larger metal particles leading to larger diameter tubes, could just as well be achieved by a pre-treatment annealing of the catalyst with growth at a lower temperature. Furthermore, we suggest that a combination of low temperature and high precursor pressure would result in the smallest diameter tubes. A combination of alloying with Mn, Mo, W, or Cr and/or a porous and sticky substrate would possibly open up for the production of exotic small diameter tubes.

## Conclusions

We have compared computed SWNT fragment stabilities from DFT with all CVD growth experiments evaluated by methods that properly detect all present nanotube chiralities. We have found that the very smallest diameter tubes of the 8, 9, and 10 series are not produced because of high formation enthalpies, but that otherwise the SWNT diameter is governed by the well-known dependence with the catalytic particle size, not by the SWNT formation enthalpy, the latter favoring large-diameter SWNTs. It is, however, natural that tubes of larger diameter than the catalytic particles are not formed. Our most important finding is that the specific chirality of the SWNT product is strongly dependent on the relative stability of the tubes within their series, which can be rationalized to a dependence on the formation enthalpy of tubes of similar diameter. Thus, the dominating SWNT products are among the few most stable in each series, and our study shows that 84% of the products in all reported CVD growth to date is within 0.2 eV of the most stable tube of its series. We wish to point out that our strong correlation between energetic stability and abundance in experimental studies is obtained by only considering the SWNT segments, without the inclusion of the interface with the catalytic metal. We thus conclude that the effect of the actual growth conditions and the interaction with the metal is less dominant with respect to the product outcome, and that the initial formation of the product is governed mainly by thermodynamic control. Regarding the possibility to influence product outcome by fine-tuning the experimental conditions, our analysis shows that the growth temperature has by far the largest effect on the index of the SWNT product. We show, however, that this is directly related to the temperature influence on the metal particle size. At low growth temperatures (below 650 °C), or high melting temperature of the catalyst due to alloying, or strong substrate binding, the “frozen” metal surfaces can act as templates for growth of less stable tubes. Such conditions account for most of the remaining 16% experimental products, and thus strengthens our already strong correlation with the fragment stability. Other parameters such as carbon precursor pressure, vapor pressure, and feed stock composition have less impact on the chirality of the product, but here we note that the low number of appropriate experimental studies makes it difficult to perform a full evaluation.

Lastly we show that the choice of substrate and catalyst composition can drastically diminish the influence of temperature on the metal particle size and lead to static/frozen metal surfaces that act as templates for growth of less stable SWNTs, and even lead to metal particle seeds for index specific growth.

## Additional Information

**How to cite this article**: Hedman, D. *et al.* On the Stability and Abundance of Single Walled Carbon Nanotubes. *Sci. Rep.* **5**, 16850; doi: 10.1038/srep16850 (2015).

## Supplementary Material

Supplementary Table S1

## Figures and Tables

**Table 1:** Energy windows (∆E) of the difference between the most stable and least stable SWNT within the series.

Series	8	9	10	11	12	13	14	15	16	17	18
∆E (eV)	2.023	1.385	0.935	0.667	0.470	1.420	1.517	2.246	2.590	3.079	3.489
