# Supplementary material for: On the Stability and Abundance of Single Walled Carbon Nanotubes
Source: Sci Rep. 2015 Nov 19;5:16850. doi: 10.1038/srep16850 (PMC4652236; doi:10.1038/srep16850)
Supplement: Supplementary Table S1 [file srep16850-s1.doc]

On the Stability and Abundance of Single Walled Carbon Nanotubes

Daniel Hedman^1,&^, Hamid Reza Barzegar^2,4,&^, Arne Rosén^3,*^, Thomas Wågberg^2,*^, J. Andreas Larsson^1,*,§^

1. Applied Physics, Division of Materials Science, Department of Engineering Sciences and Mathematics, Luleå University of Technology, SE-971 87 Luleå, Sweden.
2. Department of Physics, Umeå University, SE-901 87 Umeå, Sweden
3. Department of Physics, University of Gothenburg, SE-412 96 Göteborg, Sweden
4. Department of Physics, University of California and the Lawrence Berkeley National Laboratory, Berkeley CA 94720, USA

^&^ Equal contribution as first author.

^*^ Equal contribution as corresponding authors.

^§^ Email: andreas.1.larsson@ltu.se

Table S1: Details of the experimental work with product properly analyzed. Reference numbers according to the main manuscript.

| Ref. | Catalyst | Pre-Treat. | Substrate | Temp. | Precursor | Pressure | Dominated Product | Characterization Techniques |
| --- | --- | --- | --- | --- | --- | --- | --- | --- |
| 10 | Co/Mo | H_2_ 500 | Silica | 800 | C_2_H_5_OH | 5 mbar | (7,6), (8,6), (8,4) | PL |
| 10 | Co/Mo | H_2_ 500 | Silica | 800 | CH_3_OH | 5 mbar | (7,6), (8,6), (8,4) | PL |
| 10 | Co/Mo | H_2_ 500 | Silica | 800 | C_2_H_2_ | Amb. | (8,4), (7,6), (7,5), (8,6) | PL |
| 10 | Co/Mo | H_2_ 500 | Silica | 800 | CO | 6 bar | (7,6), (7,5), (8,4) | PL |
| 11 | Co/Mo | H_2_ 500 | Silica | 750 | CO | 5 atm | (7,5), (6,5) | PL |
| 14 | Co | Vacuum 600/700 | SiO_2_ wafer | 600 | C_2_H_2_ | 10-3 mbar | (6,5) 27%, (6,6) 19%, (7,4) 19% | Raman: 8 excitation energies between 1.96 and 2.66 eV |
| 14 | Co | Vacuum 600/700 | SiO_2_ wafer | 700 | C_2_H_2_ | 10-3 mbar | (7,5) 14%, (7,6) 11%, (10,9) 11% | Raman: 8 excitation energies between 1.96 and 2.66 eV |
| 15 | Co | Ar 600 | Silica | 600 | CO | Amb. | (6,5), (7,5) | UV-Vis-NIR and PL |
| 16 | Fe/Cu | _ | MgO | 600 | CO | Amb. | (6,5) dominant | UV-Vis-NIR and PL |
| 16 | Fe/Cu | _ | MgO | 750 | CO | Amb. | (7,5), (6,5), (8,4) | UV-Vis-NIR and PL |
| 16 | Fe/Cu | _ | MgO | 800 | CO | Amb. | (7,5), (8,4), (6,5), (7,6) | UV-Vis-NIR and PL |
| 17 | Fe | Ar 800 | Si_3_N_4_ TEM grid | 800 | CO | Amb. | (8,6), (9,7) | Electron diffraction and Raman (633 nm) |
| 17 | Fe | Ar 800 | Si_3_N_4_ TEM grid | 800 | CH_4_ | Amb. | - | Electron diffraction and Raman (633 nm) |
| 18 | Co | _ | MgO | 400 | CO | Amb./6.3 | (7,6), (9,4) | Raman and PL, UV-vis-NIR and electron diffraction |
| 18 | Co | _ | MgO | 500 | CO | Amb./6.3 | (6,5) 53% | Raman and PL, UV-vis-NIR and electron diffraction |
| 18 | Co | _ | MgO | 600 | CO | Amb./6.3 | (6,5), (7,5), (8,3) | Raman and PL, UV-vis-NIR and electron diffraction |
| 20 | Fe/Co (1:1) | Ar/H_2_ 800 | Si wafer | 800 | C_2_H_2_ | Amb. | (7,5) + (8,5) + (12,1) + (12,0) = 29.9% | Raman (488, 633, 785 nm) |
| 20 | Fe/Co (1,12:1) | Ar/H_2_ 800 | Si wafer | 800 | C_2_H_2_ | Amb. | (7,5) + (8,5) + (12,1) + (12,0) = 68.4% | Raman (488, 633, 785 nm) |
| 21 | Co | H_2_ 500 | SiO_2_ | 780 | CO | 6 bar | (6,5), (7,5), (8,4) | Raman, PL and UV-Vis-NIR |
| 35 | Co S dop | H_2_ 500 | SiO_2_ | 780 | CO | 6 bar | (9,8) 40%, (10,9), (10,6), (9,7) | Raman, PL and UV-Vis-NIR |
| 52 | Co | H_2_ 400 | Silica TUD-1 | 800 | CO | 6 bar | (6,5) 32.6% | UV-Vis-NIR, PL and Raman (785 and 633 nm) |
| 52 | Co | H_2_ 500 | Silica TUD-1 | 800 | CO | 6 bar | (9,8) 59.1% | UV-Vis-NIR, PL and Raman (785 and 633 nm) |
| 52 | Co | H_2_ 600 | Silica TUD-1 | 800 | CO | 6 bar | (6,5) 32.6%, (9,7) 12.3%, (6,5) 8.9% (10,9) 7.4% | UV-Vis-NIR, PL and Raman (785 and 633 nm) |
| 53 | Ni_x_  Fe_1-x_ | _ | Gas phase | 600, 700 | C_2_H_2_ | Amb. | more Fe, less metallic SWNTs | UV-Vis-NIR, PL and Raman (633, 514 and 488 nm) |
| 53 | Ni | _ | Gas phase | 600, 700 | C_2_H_2_ | Amb. | (9,4) dominant | UV-Vis-NIR, PL and Raman (633, 514 and 488 nm) |
| 53 | Ni_0.67_ Fe _0.33_ | _ | Gas phase | 600, 700 | C_2_H_2_ | Amb. | (7,6) dominant | UV-Vis-NIR, PL and Raman (633, 514 and 488 nm) |
| 53 | Ni_0.5_  Fe_0.5_ | _ | Gas phase | 600, 700 | C_2_H_2_ | Amb. | (8,4) dominant | UV-Vis-NIR, PL and Raman (633, 514 and 488 nm) |
| 53 | Ni_0.27_  Fe_0.73_ | _ | Gas phase | 600, 700 | C_2_H_2_ | Amb. | (8,4) dominant | UV-Vis-NIR, PL and Raman (633, 514 and 488 nm) |
| 54 | CoSO_4_ | H_2_ 540 | SiO_2_ | 780 | CO | 6 bar | (9,8) 33.5%, (9,7) 7.1% | Raman (785, 633, 514 nm), PL and UV-Vis-NIR |
| 54 | CoSO_4_ | H_2_ 780 | SiO_2_ | 780 | CO | 6 bar | - | Raman (785, 633, 514 nm), PL and UV-Vis-NIR |
| 55 | Fe | Ar 750 | MgO porous | 750 | CH_4_ | Amb. | (8,5), (7,5), (7,6), (8,6), (8,4), (9,4) | Raman (514.5 nm), PL and UV-Vis-NIR |
| 55 | Fe | Ar 750 | MgO porous | >750 | CH_4_ | Amb. | Broader diameter distribution | Raman (514.5 nm), PL and UV-Vis-NIR |
| 56 | Co/Mo | H_2_ 500 | Silica | 700 | CO | Amb. | (6,5) 54%, (6,6) 23%, (7,7) 13% | PL and UV-Vis-NIR |
| 56 | Co/Mo | H_2_ 500 | Silica | 750 | CO | Amb. | (6,5) 42%, (6,6) 19%, (7,7) 13%, (8,4) 16% | PL and UV-Vis-NIR |
| 56 | Co/Mo | H_2_ 500 | Silica | 800 | CO | Amb. | (6,5) 55%, (6,6) 15%, (7,7) 10% | PL and UV-Vis-NIR |
| 56 | Co/Mo | H_2_ 500 | Silica | 850 | CO | Amb. | (6,6) 17%, (7,6) 15%, (7,7) 14%, (8,7) 13% | PL and UV-Vis-NIR |
| 56 | Co/Mo | H_2_ 350 | MgO | 750 | CO | Amb. | (7,5) 26%, (6,6) 22%, (6,5) 19% | PL and UV-Vis-NIR |
| 57 | Co/Mo (1:3) | H_2_ 500 | Silica | 800 | CO | 2 bar | (7,6) 26%, (8,6), (9,4), (6,5) 2% | UV-vis-NIR, optical abs. and Raman (514, 633, 785 nm) |
| 57 | Co/Mo (1:3) | H_2_ 500 | Silica | 800 | CO | 12 bar | (7,5) 28%, (6,5), (7,6), (8,3) | UV-vis-NIR, optical abs. and Raman (514, 633, 785 nm) |
| 57 | Co/Mo (1:3) | H_2_ 500 | Silica | 800 | CO | 18 bar | (6,5) 48%, (8,3), (7,5), (7,6) 7% | UV-vis-NIR, optical abs. and Raman (514, 633, 785 nm) |
| 69 | W/Co | H_2_ | Si wafer | 1030 | Ethanol | Amb. | (12,6) 92% | Surface-enhanced Raman and UV-vis-NIR, optical abs. |
| 78 | Co | NH_3_ | Silicon wafer | 700 | C_2_H_2_ | 10-3 mbar | (6,5), (7,5), (9,2) | Raman: 8 excitation energies between 1.96 and 2.66 eV |
| 78 | Co | vacuum | Silicon wafer | 700 | C_2_H_2_ | 10-3 mbar | (7,5), (7,6), (10,9) | Raman: 8 excitation energies between 1.96 and 2.66 eV |
| 78 | Co | NH_3_ | Silicon wafer | 700 | C_2_H_5_OH | 5 mbar | (7,6), (7,7), (10,9) | Raman: 8 excitation energies between 1.96 and 2.66 eV |
| 78 | Co | vacuum | Silicon wafer | 700 | C_2_H_5_OH | 5 mbar | (14,9), (9,8), (10,9) | Raman: 8 excitation energies between 1.96 and 2.66 eV |
| 79 | Fe 60%, Ni 11%, Cr 18% | Calcinated at 700 | Stainless steel wire | 700 | CO | Amb. | (6,5), (7,5), (8,3) | PL and Raman |
| 80 | Fe/Ru | _ | Silica | 600 | CH_4_ | Amb. | (6,5) dominant | PL |
| 80 | Fe/Ru | _ | Silica | 700 | CH_4_ | Amb. | (6,5), (7,6), (8,4) | PL |
| 80 | Fe/Ru | _ | Silica | 850 | CH_4_ | Amb. | (7,5), (7,6), (8,4) | PL |
| 81 | Co | H_2_ 750 | C10-MCM-41 | 550 | CO | Amb. | (6,5), (8,4) | Raman (488, 532, 785 nm), PL, UV-Vis-NIR |
| 81 | Co | H_2_ 750 | C10-MCM-41 | 650 | CO | Amb. | (6,5), (7,5), (7,6), (8,4) | Raman (488, 532, 785 nm), PL, UV-Vis-NIR |
| 81 | Co | H_2_ 750 | C10-MCM-41 | 750 | CO | Amb. | (7,5), (7,6), (8,4), (8,6) | Raman (488, 532, 785 nm), PL, UV-Vis-NIR |
| 81 | Co | H_2_ 750 | C10-MCM-41 | 950 | CO | Amb. | (7,6), (8,4), (8,6) + wide distribution of other tubes | Raman (488, 532, 785 nm), PL, UV-Vis-NIR |
| 82 | Co/Fe | Ar/H_2_ 650 | Zeolite | 650 | C_2_H_5_OH | 10 Torr | (6,5), (7,5) | Optical abs., NIR fluorescence and Raman (488 nm) |
| 82 | Co/Fe | Ar/H_2_ 750 | Zeolite | 750 | C_2_H_5_OH | 10 Torr | (7,5), (6,5) | Optical abs., NIR fluorescence and Raman (488 nm) |
| 82 | Co/Fe | Ar/H_2_ 850 | Zeolite | 850 | C_2_H_5_OH | 10 Torr | (7,5), (7,6), (8,6) | Optical abs., NIR fluorescence and Raman (488 nm) |
| 83 | Y/Ni | no mag. field |  | arc temp | amorph C | 66.5 KPa | - | Raman (514 nm), PL and UV-Vis-NIR |
| 83 | Y/Ni | magnetic field |  | arc temp | Amorph C | 66.5 KPa | (7,6), (8,6), (10,3), (8,7) | Raman (514 nm), PL and UV-Vis-NIR |
| 85 | Co | H_2_ 500 | MCM-41 | 675 | C_2_H_5_OH | 0.1 mbar | (6,5), (7,3) | PL and UV-Vis-NIR |
| 85 | Co | H_2_ 500 | MCM-41 | 725 | C_2_H_5_OH | 0.1 mbar | (6,5), (7,5), (7,3) | PL and UV-Vis-NIR |
| 85 | Co | H_2_ 500 | MCM-41 | 750 | C_2_H_5_OH | 0.1 mbar | (6,5), (7,5) | PL and UV-Vis-NIR |
| 85 | Co | H_2_ 500 | MCM-41 | 775 | C_2_H_5_OH | 0.1 mbar | (7,5), (8,4), (6,5) | PL and UV-Vis-NIR |
| 85 | Co | H_2_ 500 | MCM-41 | 800-825 | C_2_H_5_OH | 0.1 mbar | (7,5), (8,4), (7,6) | PL and UV-Vis-NIR |
| 86 | Co/Mn (1:3) | H_2_ 700 | MCM-41 | 600 | CO | Amb. | (6,5) 40%, (8,3) 20.4%, (7,3) 18.7% | PL and Raman (7 different wavelength from 488 - 1064) |
| 86 | Co/Mn (1:3) | H_2_ 700 | MCM-41 | 700 | CO | Amb. | (6,5) 45%, (7,3) 16,4%, (8,3) 13.4% | PL and Raman (7 different wavelength from 488 - 1064) |
| 86 | Co/Mn (1:3) | H_2_ 700 | MCM-41 | 800 | CO | Amb. | (6,5) 40%, (7,5) 20.9% | PL and Raman (7 different wavelength from 488 - 1064) |
| 88 | Fe | _ | Gas phase | 880 | CO, CO_2_ | Amb. | (12,10), (11.9), (12,12) | Electron diffraction |
| 88 | Fe | _ | Gas phase | 880 | CO, CO_2_ | Amb. | (13,12), (12,11), (13,11) | Electron diffraction |
| 89 | Molecular precursor | - | Pt | 400-500 | Ethylene /Ethanol | 10^-7^ mbar | (6,6) 100% | Raman |
| 103 | W/Co | - | SiO_2_ | 1050 | Ethanol | Amb. | (16,0) 80% | PL and Raman |
